# CLEC4G Reverses Lenvatinib Resistance in Hepatocellular Carcinoma by Suppressing PD‐1 Expression via the Wnt/*β*‐Catenin Pathway

**DOI:** 10.1155/ijog/9225945

**Published:** 2025-12-11

**Authors:** Kemin Xiao, Jin Yan, Guangxi He, Bin He, Qi Wang

**Affiliations:** ^1^ Department of General Surgery, Daye People’s Hospital, Daye, Hubei, China

**Keywords:** bioinformatics analysis, *CLEC4G*, drug resistance, hepatocellular carcinoma, immune escape, lenvatinib

## Abstract

**Objective:**

The objective was to analyze the effect of *C-type lectin domain family 4 member G* (*CLEC4G*) on hepatocellular carcinoma (HCC) and investigate its impact on lenvatinib (Lenva) resistance as well as the underlying action pathway.

**Methods:**

Differentially expressed genes (DEGs) were screened from the GSE101685 dataset, followed by functional enrichment analysis. Subsequently, *CLEC4G* was selected for subsequent experiments. The Lenva‐resistant cell line PLC/PRF/5‐R was established and transfected with a *CLEC4G* silencing expression vector to observe alterations in its biological behavior. Sample size was estimated based on a pilot experiment (*n* = 3 biological replicates) with 30 clinical samples/cell experiment in each group. Additionally, the expression of the Wnt/*β*‐catenin pathway in PLC/PRF/5‐R was examined, and PLC/PRF/5‐R activity after intervention with LiCl, a Wnt/*β*‐catenin pathway activator, was evaluated.

**Results:**

A total of 51 DEGs were identified in the GSE101685 dataset. After silencing CLEC4G expression, the activities of both PLC/PRF/5 and PLC/PRF/5‐R, as well as the expression of PD‐1, were decreased, while apoptosis was increased (*p* < 0.05). Moreover, silencing CLEC4G inhibited the expression of the Wnt/*β*‐catenin pathway (*p* < 0.05). After LiCl intervention, the activity of PLC/PRF/5‐R was enhanced, and the expression of PD‐1 was elevated (*p* < 0.05). Silencing CLEC4G could reverse the effect of LiCl on PLC/PRF/5‐R.

**Conclusion:**

*CLEC4G* modulates the *PD-1* expression of HCC cells through the Wnt/*β*‐catenin pathway, thereby reversing the resistance to Lenva.

## 1. Introduction

Hepatocellular carcinoma (HCC), being the most common type of primary liver cancer and one of the malignancies with the highest mortality rates globally, is characterized by latent onset, high‐level malignancy, and a predisposition to metastasis and recurrence [1]. According to global epidemiological statistics, there were approximately 367,700 new HCC cases and around 316,500 deaths in 2022, ranking only second to lung cancer in terms of severity [2]. Currently, the dearth of specific early diagnosis markers for HCC in clinical practice has resulted in over 60% of HCC patients being diagnosed at the intermediate‐to‐advanced stage and missing the optimal time window for surgical resection [3]. Lenvatinib (Lenva) is a molecular targeted drug for unresectable HCC. As a novel small‐molecule inhibitor of tyrosine kinase, Lenva was approved by the Food and Drug Administration as a first‐line targeted therapy for advanced HCC in 2018 [4]. Currently, clinical studies have demonstrated that the median survival period of HCC patients after Lenva treatment surpasses that of those treated with sorafenib [5], which has ushered in new hope for the future treatment of HCC. Nevertheless, drug resistance in molecular targeted drugs is inevitable, which has emerged as a critical issue demanding urgent resolution in modern clinical research.

Immune escape is considered one of the significant mechanisms of drug resistance. Tumor cells can evade the recognition of the immune system by suppressing antigen presentation, hiding or altering self‐antigens, and so forth, thus generating resistance to therapeutic drugs [6]. Consequently, a growing body of studies has proposed that intervening in cellular immune escape may be a breakthrough in preventing and reversing tumor drug resistance [6, 7]. For HCC, there are also some studies proposing potential immune escape mechanisms. For example, Ruiz de Galarreta et al. found that *β*‐catenin activation promoted immune escape and resistance to anti‐programmed death‐1 (PD‐1) treatment in HCC [8]. Recently, in Wan and Zhu′s study, they reported a successful case of using Regorafenib combined with Sintilimab (PD‐1 inhibitor) as a second‐line treatment for advanced HCC patients [9]. This provides a solid foundation for PD‐1 targeted intervention to reverse Lenva resistance. However, the mechanisms by which PD‐1 reverses Lenva resistance are not fully understood. This is also the difficulty of modern research.

In light of this circumstance, this research will utilize online databases to screen the potential differentially expressed genes (DEGs) in HCC and further conduct an in‐depth exploration of the influence of these genes on the PD‐1 expression of HCC and resistance to Lenva, with a view to providing new ideas for the clinical prevention and reversal of HCC′s resistance to Lenva in the future and enhancing the prognosis of HCC patients.

## 2. Materials and Methods

### 2.1. Main Reagents and Instruments

Human HCC cells PLC/PRF/5 and the supporting medium (SNLM‐086) were purchased from Wuhan Shanen Biotechnology Co. CCK‐8 kit was purchased from Shanghai Biyuntian Biotechnology Co (c0041). Lipofectamine^3000^ transfection reagent (L3000008), TRIpure Reagent (17926), SYBR Green I (S7563), and sodium dodecyl sulfate‐polyacrylamide gel electrophoresis (SDS‐PAGE) gel (89888) were purchased from Thermo Fisher, United States. Western blot antibody (*CLEC4G*: ab181196, *β-catenin*: ab32572, *c-myc*: ab32072, *Cyclin-D1*: ab134175, and GAPDH: ab8245) was purchased from Abcam (United Kingdom); RIPA lysate (V900854), Annexin V‐FITC (APOAF), and PI (850181P) were purchased from Sigma‐Aldrich (Shanghai); Transwell (CLS3401) was purchased from Corning (United States); Wnt/*β*‐catenin pathway activator LiCl (PNU‐74654) was purchased from Shanghai Sangong Bioengineering Co. CytoFLEX LX flow cytometer, Beckman Coulter, United States; Varioskan LUX enzyme labeling instrument, Thermo Fisher, United States; LSM800 laser confocal microscope, Zeiss, United States; and ExicyclerTM 96 polymerase chain reaction (PCR) instrument, Bioneer, Korea.

### 2.2. Dataset Source

GSE101685 from the Gene Expression Omnibus (GEO) database (https://www.ncbi.nlm.nih.gov/), including eight normal liver tissues and 24 liver cancer tissues, was selected for analysis. The analysis platform used was GPL570 [HG‐U133_Plus_2] Affymetrix Human Genome U133 Plus 2.0 Array.

### 2.3. DEG Screening and Functional Enrichment Analysis

The Gene Expression Omnibus to R (GEO2R) plug‐in in the GEO database was used to analyze the cancerous and adjacent normal tissue samples in the GSE101685 dataset. After removing duplicate and named DEGs, the GEO2R default parameters were used: adj.p.valr < 0.05 (Benjamini–Hochberg correction), |logFC| > 4 was the difference threshold, and the volcano plot was generated based on the ggplot2 package. Using the Kyoto Encyclopedia of Genes and Genomes (KEGG) and Gene Ontology (GO) annotations in the R software package org.Hs.db as the background, the DEGs were mapped to the background set, and the analysis results were presented with false discovery rate (FDR) < 0.05 and *p* < 0.05.

### 2.4. Bioinformatics Analysis

We downloaded the standardized pan‐cancer dataset from the UCSC database (https://xenabrowser.net/): TCGA TARGET GTEx (PANCAN, *N* = 19,131, *G* = 60,499). Subsequently, *C-type lectin domain family 4 member G* (*CLEC4G*) expression data, prognostic data, and immune checkpoint pathway genes in each sample were extracted and converted by Log2 (*x*+0.001), followed by plotting the expression and prognostic impact of *CLEC4G* in multiple tumors and analyzing the correlation between *CLEC4G* and marker genes of the five types of immune pathways by Pearson correlation coefficient. The relationship between *CLEC4G* and immune cells in HCC was analyzed using the TIMER database (http://timer.cistrome.org/), and the effect of *CLEC4G* on the degree of immune cell infiltration in HCC was observed using SCNA instructions.

### 2.5. Clinical Information

We included 34 patients with HCC who received Lenva chemotherapy from January 2024 to June 2024 at our institution as study subjects. Tissue samples and paracancerous tissue samples collected at the time of pathological examination of the patients were obtained, and their peripheral blood samples before and after chemotherapy were collected for *CLEC4G* expression detection. Inclusion criteria: (1) HCC diagnosed by pathological biopsy, (2) complete clinical data, (3) unresectable advanced HCC or locally progressive stage, and (4) expected survival ≥ 3 months. Exclusion criteria: (1) presence of severe cardiovascular disease, (2) severe infection, (3) history of other malignant tumors within 5 years, (4) allergy to Lenva, and (5) pregnant or lactating women. This study was approved by the Ethics Committee of our hospital (No. KY2024‐022).

### 2.6. PCR

Total RNA was extracted by lysing the blood or tissue with TRIpure Reagent, followed by reverse transcription to obtain the corresponding complementary deoxyribonucleic acid (cDNA). Utilizing the cDNA as a template, quantitative analysis was executed using the SYBR Green method on an ExicyclerTM 96 real‐time fluorescence quantitative PCR instrument. The expression level of *CLEC4G* mRNA relative to *β*‐actin was calculated using the 2^- ≥ ≥Ct^ method [10]. The primer sequences are shown in Table 1.

**Table 1:** Sequence of primers.

	**F**	**R**
*CLEC4G*	5 ^′^‐ACAGTCCTTTGGGCTGTGAT‐3 ^′^	5 ^′^‐TTTGTCCTCAGCAGGTCGT‐3 ^′^
*GAPDH*	5 ^′^‐GGAGCGAGATCCCTCCAAAAT‐3 ^′^	5 ^′^‐GGCTGTTGTCATACTTCTCATGG‐3 ^′^

### 2.7. Cell Culture

PLC/PRF/5 cells were cultured in Dulbecco′s modified eagle medium (DMEM) supplemented with 10% fetal bovine serum and 1% penicillin‐streptomycin in a 30°C, 5% CO₂ incubator (this cell line is a model of HBV‐related liver cancer, and low‐temperature culture can maintain viral antigen expression). When the cell confluence reached 80%, the cells were passaged by trypsin digestion (every 2–3 days). Cells in the logarithmic growth phase were collected for subsequent experiments.

### 2.8. Construction of Lenva‐Resistant Cells

**Figure 1:**
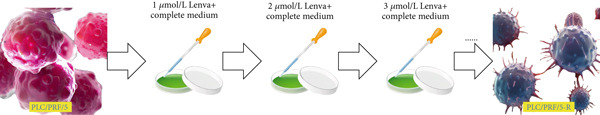
Construction process of Lenva‐resistant cells.

PLC/PRF/5 cells in optimal growth state were selected for the construction of Lenva‐resistant cells through the gradient–concentration–induction approach. When the cells grew and fused to 50%–60%, they were cultured in a complete medium supplemented with 1 *μ*mol/L of Lenva for a period of 24 h. Subsequently, the medium was replaced with a standard complete medium. Subculturing was performed once the cells reached confluence (repeated twice). Thereafter, the Lenva concentration was adjusted to 2 *μ*mol/L, and the aforementioned procedures were reiterated until the Lenva concentration reached 10 *μ*mol/L. The resultant Lenva‐resistant cells were designated as PLC/PRF/5‐R. To maintain the drug‐resistant characteristics of PLC/PRF/5‐R, these cells were cultured in a complete medium containing 10 *μ*mol/L of Lenva (Figure 1).

### 2.9. Verification of the PLC/PRF/5‐R Model

PLC/PRF/5 and PLC/PRF/5‐R, which were in a good growth state and during the logarithmic growth phase, were carefully selected and inoculated into 96‐well plates at a density of 5000 cells per well. Upon 60%–70% confluence, they were divided into a control group (containing culture medium but without drugs) and multiple gradient drug intervention groups (each containing medium along with different concentrations of Lenva, namely, 2, 4, 8, 16, 32, 64, 128, and 256 *μ*mol/L). Concurrently, a blank group devoid of both cells and drugs was established. Each group was provided with six multiple wells. After 24 h of incubation, the medium was discarded, and CCK‐8 was added at 10 *μ*L/well. Following incubation for 1 h, the optical density (OD) of each well (450 nm) was detected using a microplate reader. The half‐maximal inhibitory concentration (IC_50_), cell inhibition rate (calculated using the formula: [1 − (OD of the experimental group − OD of the blank group)/(OD of the control group − OD of the blank group)] × 100*%*), and resistance index (IC_50_ of resistant cells divided by IC_50_ of parental cells) were then computed.

### 2.10. Cell Transfection

Short hairpin ribonucleic acid (shRNA) interference fragments and corresponding negative controls specific to *CLEC4G* were synthesized, constructed, and then transfected into the logarithmic‐growth‐phase cells following the instructions of Lipofectamine^3000^. The experimental groups are detailed as follows: sh‐CLEC4G group: PLC/PRF/5 cells were transfected with CLEC4 interference fragments; sh‐NC group: PLC/PRF/5 cells were transfected with shRNA interference control fragments; sh‐CLEC4G‐R group: PLC/PRF/5‐R cells were transfected with CLEC4 interference fragments; sh‐NC‐R group: PLC/PRF/5‐R cells were transfected with shRNA interference control fragments. After transfection, real‐time fluorescence quantitative PCR was employed to assess the transfection efficiency.

### 2.11. Cell Cloning

Cells were inoculated into 6‐well plates at a density of 800 cells per well, and the medium was replaced every 2 days. After a 2‐week period, they were fixed with 4% paraformaldehyde for 1 h and then stained with 0.1% crystal violet at room temperature for 30 min. Subsequently, the number of colonies formed was counted, and the cell proliferation rate was calculated.

### 2.12. Apoptosis

Cells were seeded in a 96‐well plate at a concentration of 5 × 10^4^/mL (200 *μ*L per well), six compound holes were set in each group, and the culture medium was discarded 12 h later. Following washing once with phosphate‐buffered saline (PBS), the cells were placed into a working trypsin solution (without ethylenediaminetetraacetic acid (EDTA)) for digestion. After centrifugation, the supernatant was discarded, and the cells were washed twice with PBS. Then, 195 *μ*L of Annexin V‐FITC binding buffer was added to resuspend the cells. Next, 5 *μ*L of Annexin V‐FITC and 10 *μ*L of propidium iodide (PI) staining solution were added. Finally, the apoptosis rate of the cells was detected using a flow cytometer.

### 2.13. Transwell

Then, 50 *μ*L of Matrige (15 *μ*g/*μ*L) was added to the upper chamber of the Transwell chamber to form a gel. Then, the cells were made into a single‐cell suspension with serum‐free medium for addition into the upper chamber, while a medium containing 10% fetal bovine serum was placed into the lower chamber. After 24 h, the transmembrane cells were fixed with 4% paraformaldehyde for 10 min and then stained with 0.1% crystal violet for 10 min. After that, the cells on the upper surface of the membrane were removed with a cotton swab, pictures were taken under a microscope, and the number of transmembrane cells was counted.

### 2.14. Western Blot

Cells in good growth condition and during the logarithmic growth phase were selected and seeded in 6‐well plates at a density of 5 × 10^5^ cells per well. After 15 min of cell lysis using a protein phosphatase inhibitor‐containing RIPA lysate, the cells were centrifuged at 12,000 r/min and 4°C for 15 min to obtain the protein supernatant. Then, an appropriate amount of loading buffer was added, and the samples were heated in a 100°C metal bath for 10 min for denaturation. Thereafter, 10 *μ*g of the denatured protein was separated by SDS‐PAGE, transferred onto a polyvinylidene fluoride (PVDF) membrane, and added with primary antibodies against *β-catenin*, *c-myc*, *Cyclin-D1*, and *GAPDH* (1:1000) for overnight incubation at 4°C. After TBST washing, the membrane was added with corresponding secondary antibodies (1:5000) and incubated for 1 hour at room temperature. After enhanced chemiluminescence (ECL) development and imaging, the grayscale values of the protein bands were analyzed using ImageJ software.

### 2.15. Wnt/*β*‐Catenin Pathway Intervention

Then, 10 mmol/L of LiCl was added to the medium of PLC/PRF/5‐R cells to activate the expression of the Wnt/*β*‐catenin pathway (LiCl intervention concentration was based on previous studies, which effectively activated the Wnt/*β*‐catenin pathway without cytotoxicity [11]), which was labeled as the LiCl group. In addition, normally cultured PLC/PRF/5‐R cells were set as the control group, and sh‐CLEC4G‐R cells cultured with LiCl were set as the sh‐CLEC4G‐R + LiCl group. The expression of the Wnt/*β*‐catenin pathway in each group of cells was detected by Western blot to verify the intervention effect of the activator. Cell cloning, apoptosis, and invasion were detected as above.

### 2.16. Fluorescence Staining

Cells of the control, sh‐CLEC4G‐R, LiCl, and sh‐CLEC4G‐R + LiCl groups were seeded in confocal dishes at a density of 2.5 × 10^5^ cells per mL and fixed with 4% paraformaldehyde for 15 min. Then, 0.1% Triton X‐100 was added to permeabilize them at room temperature for 10 min, followed by room temperature sealing for 60 min. Subsequently, the anti‐*PD-1* primary antibody was added to incubate overnight at 4°C (12 h). After that, the cells were incubated with an Alexa Fluor 647‐labeled fluorescent secondary antibody for 1 h at 37°C and then cultured with 4 ^′^,6‐diamidino‐2‐phenylindole (DAPI) for 10 min in the dark. After washing with PBST three times, the fluorescence intensity of *PD-1* was observed under an optical microscope.

### 2.17. Statistical Methods

R 4.1.3 software was used for statistical analysis of the data. The independent sample *t*‐test was adopted for the comparison between groups. Paired *t*‐test was used for intragroup comparison. For the comparison among multiple groups of data, repeated‐measures analysis of variance and Bonferroni within‐group test were used. Kaplan–Meier (KM) analysis was employed to evaluate the survival differences. *p* < 0.05 was used as the significance level in this study. The R software packages used for plotting included ggplot2, ggsignif, enrichplot, survival, clusterProfiler, and pRRophetic.

## 3. Results

### 3.1. DEG Analysis

Through GEO2R analysis, we found 51 DEGs from the GSE101685 dataset, of which 11 were downregulated and 40 were upregulated. The heat map showed that 51 DEGs were expressed in cancer/paracancerous tissues, and the blue cluster area suggested that genes such as CLEC4G were significantly up‐regulated (Figure 2a). The PPI network composed of these DEGs had 40 nodes and 47 edges, and no obvious core DEGs were seen. Among them, *CLEC4G*, *OIT3*, *CLEC1B*, and so forth formed a relatively stable and independent signaling pathway (Figure 2b).The results of GO analysis showed that the keywords mainly involved in these DEGs were alcohol dehydrogenase (NAD) activity, ethanol oxidation, terpenoid metabolic process, multiorganism process, and so forth (Figure 2c). We further screened the DEGs related to cellular immune function and found that all of them contained *CLEC4G* (Table 2 demonstrates all GO‐enriched functions of *CLEC4G*). KEGG analysis showed that the keywords mainly involved in these DEGs were retinol metabolism, drug metabolism‐cytochrome P450, metabolism of xenobiotics by cytochrome P450, and chemical carcinogenesis (Figure 2d).

Figure 2Results of DEGs analysis. (a) Analysis of DEGs in GSE101685 by GEO2R. (b) PPI network of DEGs. (c) Results of GO analysis of DEGs. (d) Results of KEGG analysis of DEGs.

**Figure:**
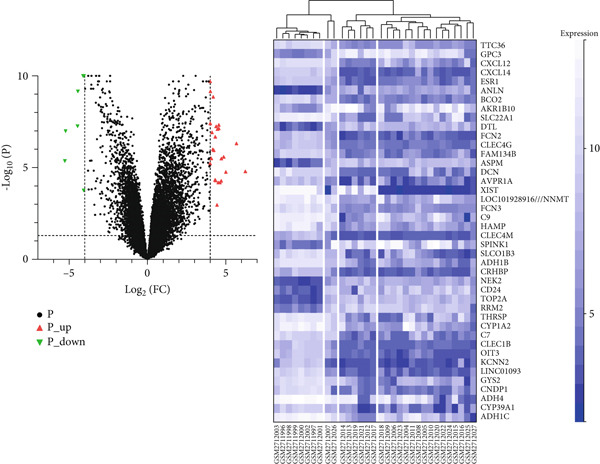
(a)

**Figure:**
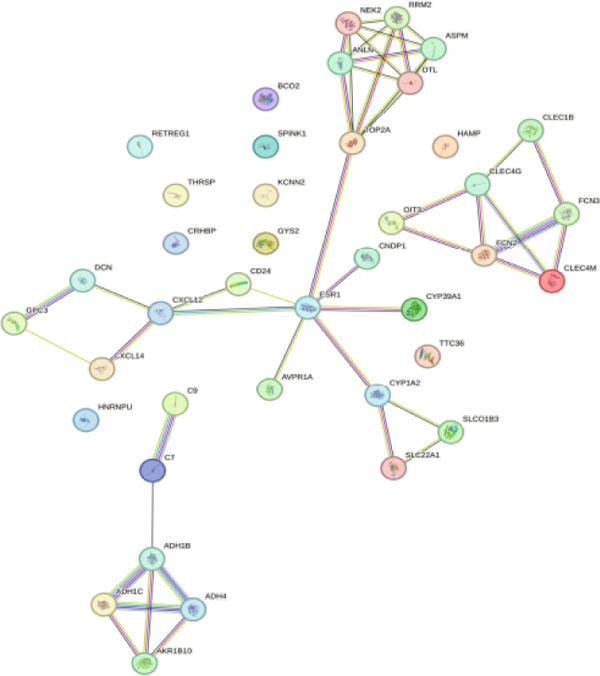
(b)

**Figure:**
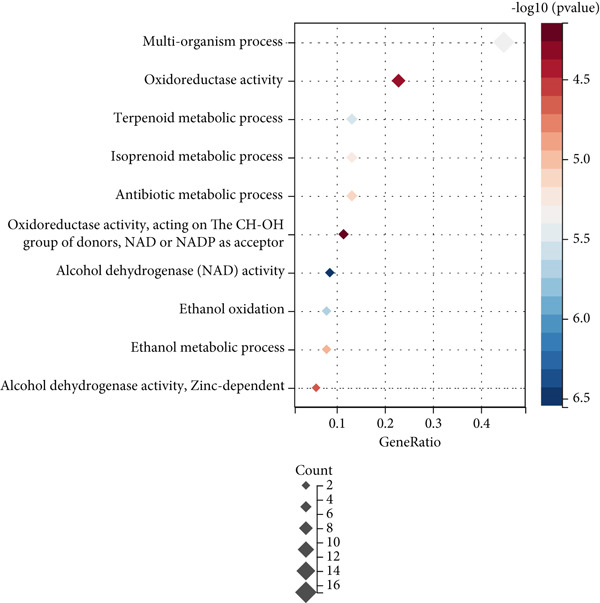
(c)

**Figure:**
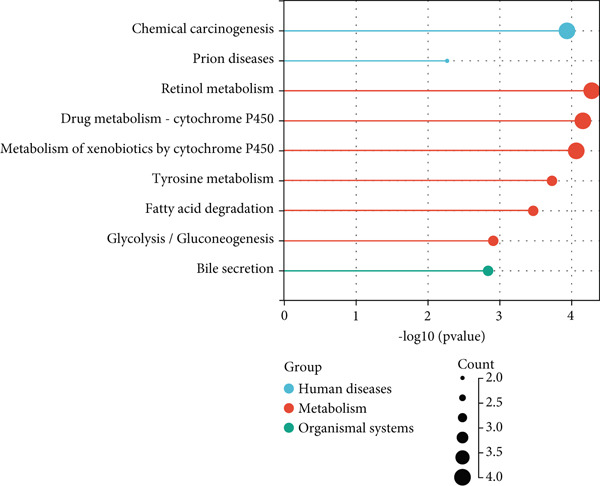
(d)

**Table 2:** Results of GO analysis for *CLEC4G.*

**ID**	**Description**	**GeneRatio**	*p*	**geneID**	**Count**	**ONT**
GO:0051704	Multi‐organism process	17/38	0.000004135572	*SPINK1/GPC3/TOP2A/ASPM/CD24/CRHBP/AVPR1A/CLEC4G/DCN/CXCL12/THRSP/CLEC4M/ESR1/HAMP/FCN2/CYP1A2/FCN3*	17	BP
GO:0030246	Carbohydrate binding	5/35	0.000156651	*CLEC4G/CLEC4M/FCN2/CLEC1B/FCN3*	5	MF
GO:0001871	Pattern binding	2/35	0.000847004	*CLEC4G/FCN2*	2	MF
GO:0030247	Polysaccharide binding	2/35	0.000847004	*CLEC4G/FCN2*	2	MF
GO:0002376	Immune system process	15/38	0.000895463	*GPC3/TOP2A/CD24/ANLN/CLEC4G/CXCL12/C7/C9/CLEC4M/ESR1/HAMP/FCN2/CLEC1B/CXCL14/FCN3*	15	BP
GO:0001618	Virus receptor activity	2/35	0.010173816	*CLEC4G/CLEC4M*	2	MF
GO:0104005	Hijacked molecular function	2/35	0.010173816	*CLEC4G/CLEC4M*	2	MF
GO:0046718	Viral entry into host cell	3/38	0.002135506	*CLEC4G/CLEC4M/FCN3*	3	BP
GO:0030260	Entry into host cell	3/38	0.002852943	*CLEC4G/CLEC4M/FCN3*	3	BP
GO:0044409	Entry into host	3/38	0.002852943	*CLEC4G/CLEC4M/FCN3*	3	BP
GO:0051806	Entry into cell of other organism involved in symbiotic interaction	3/38	0.002852943	*CLEC4G/CLEC4M/FCN3*	3	BP
GO:0051828	Entry into other organism involved in symbiotic interaction	3/38	0.002852943	*CLEC4G/CLEC4M/FCN3*	3	BP
GO:0006955	Immune response	11/38	0.003956584	*CD24/CLEC4G/CXCL12/C7/C9/CLEC4M/ESR1/HAMP/FCN2/CXCL14/FCN3*	11	BP

### 3.2. *CLEC4G* Exhibits Pan‐Cancer Expression Patterns and Immune Associations in HCC

First, we analyzed the expression of *CLEC4G* in various tumor diseases, and after normalization by the pan‐cancer dataset, it was seen that *CLEC4G* was aberrantly expressed in a variety of tumor diseases, with only head and neck squamous cell carcinoma (HNSC), Wilms tumor (WT), uterine carcinosarcoma (UCS), and pheochromocytoma and paraganglioma (PCPG) in which no differential expression was seen (Figure 3a). Further observation of its pathological staging in different tumors showed that *CLEC4G* was associated with breast invasive carcinoma (BRCA), stomach and esophageal carcinoma (STES), pan‐kidney cohort (KIPAN), stomach adenocarcinoma (STAD), kidney renal clear cell carcinoma (KIRC), testicular germ cell tumors (TGCT), and bladder urothelial carcinoma (BLCA) showed a significant pathological staging association (Figure 3b). In the prognostic analysis, on the other hand, we saw a significant effect of *CLEC4G* only on the prognosis of lung adenocarcinoma (LUAD) (Figure 3c). In the analysis of the immune microenvironment, we found that although there was no significant relationship between *CLEC4G* and the immune infiltration score of HCC (Figure 3d), it affected the expression of several immune checkpoints and regulatory genes in HCC (Figure 3e). Meanwhile, a synergistic effect of *CLEC4G* with CD4^+^ T cells and macrophages in HCC was seen in the TIMER database (Figure 3f). However, different copy fractions of *CLEC4G* were not seen to have an effect on the infiltration capacity of immune cells in SCNA analysis (Figure 3g).

Figure 3Results of bioinformatics analysis. (a) *CLEC4G* is aberrantly expressed in multiple cancers. (b) *CLEC4G* correlates with advanced pathological stages in specific cancers. (c) High *CLEC4G* predicts poor prognosis in lung adenocarcinoma. (d) *CLEC4G* shows no direct link to immune infiltration scores in HCC. (e) *CLEC4G* associates with immune checkpoint and regulatory genes in HCC. (f) *CLEC4G* synergizes with CD4+ T cells and macrophages in the HCC microenvironment. (g) *CLEC4G* copy number alterations do not affect immune cell infiltration.

**Figure:**
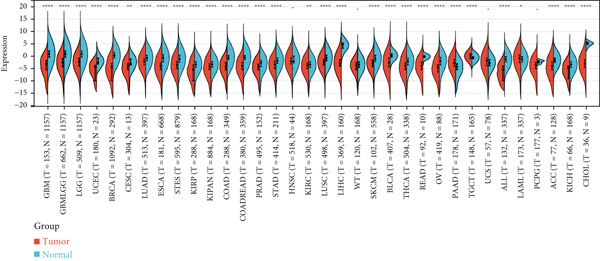
(a)

**Figure:**
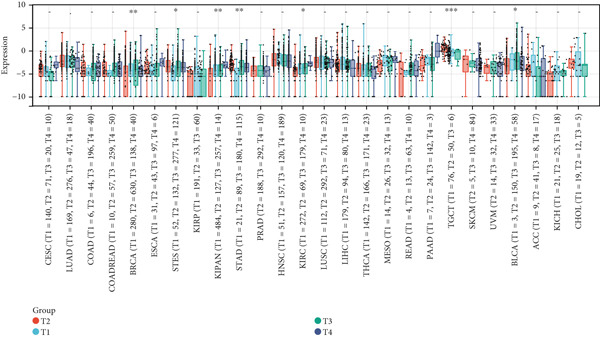
(b)

**Figure:**
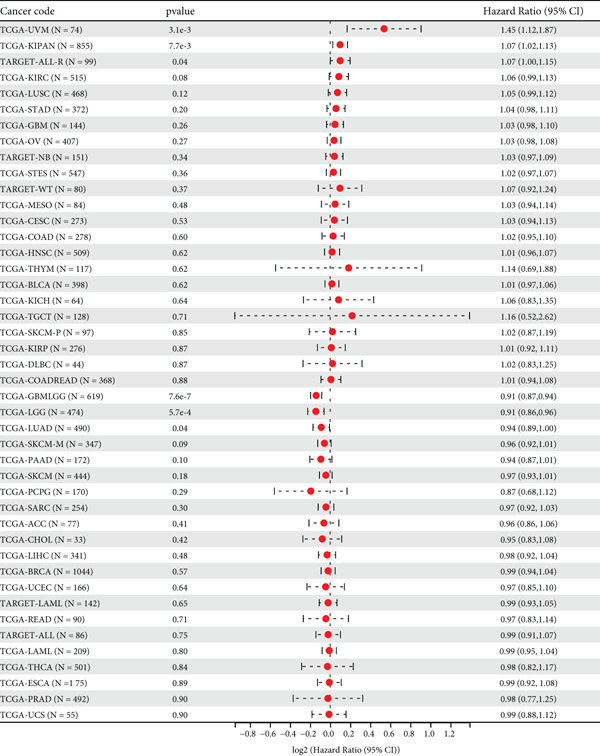
(c)

**Figure:**
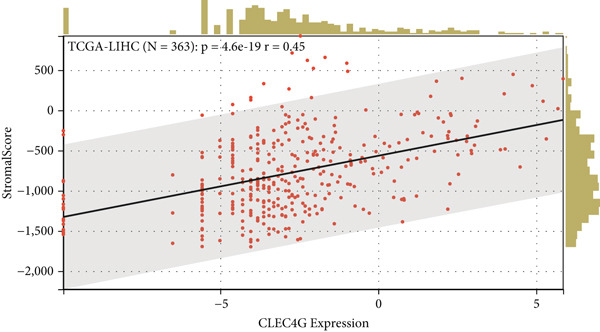
(d)

**Figure:**
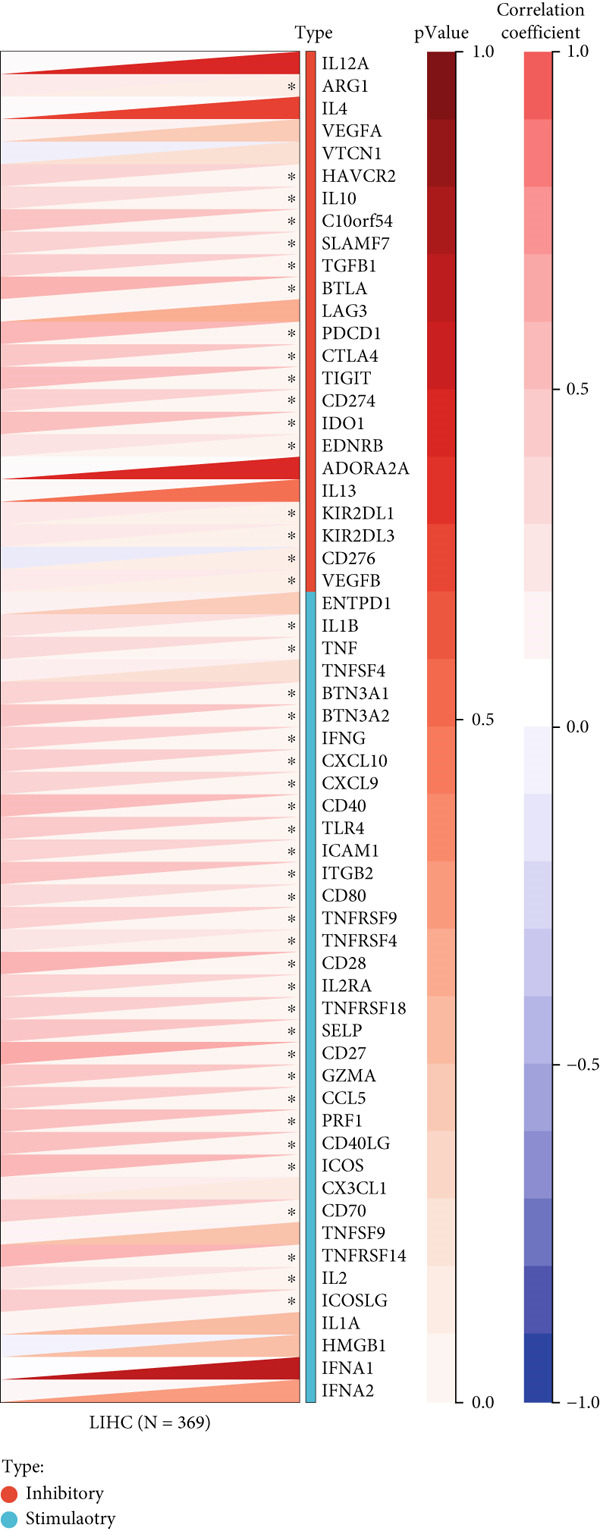
(e)

**Figure:**
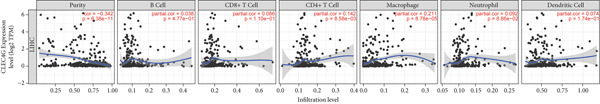
(f)

**Figure:**
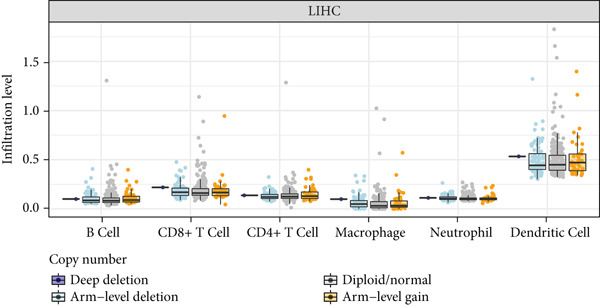
(g)

### 3.3. *CLEC4G* Silencing Inhibits HCC Cell Proliferation and Invasion

In clinical samples, *CLEC4G* mRNA expression was seen to be higher in cancer tissues than in paracancerous tissues of HCC patients (*p* < 0.01, Figure 4a), validating the results of the analysis of the appealing biological information. In addition, we also found that *CLEC4G* mRNA expression in the peripheral blood of patients was lower after chemotherapy compared with before chemotherapy (*p* < 0.01, Figure 4b). Next, we constructed lenva‐resistant cells were constructed by the gradient‐concentration method. After testing, the IC_50_ of Lenva for PLC/PRF/5 was determined to be 34.16 ± 5.04 * μ*mol/L, while for PLC/PRF/5‐R, it was 150.46 ± 14.63 * μ*mol/L, with a resistance index of 4.64 ± 0.87, verifying the successful establishment of the resistant cells (Figure 4c). After transfection with *CLEC4G* abnormal expression vectors, lower *CLEC4G* mRNA expression was determined in the sh‐CLEC4G group versus the sh‐NC group and in the sh‐CLEC4G‐R group versus the sh‐NC‐R group (*p* < 0.05), confirming the success of transfection (Figure 4d). Upon examination, the IC50 of the sh‐CLEC4G‐R group was seen to be 49.16 ± 6.03 * μ*mol/L, which was significantly lower than that of the sh‐RNA‐R group, suggesting that our silencing of *CLEC4G* expression improved the sensitivity of PLC/PRF/5‐R to Lenva (Figure 4e). In terms of biological behavior, both the cell proliferation rate and the number of invasive cells in the sh‐CLEC4G group were decreased compared to the sh‐NC group, while the apoptosis rate was elevated (*p* < 0.05, Figures 4f, 4g, and 4h). Likewise, in the sh‐CLEC4G‐R group, the cell proliferation rate and the number of invasive cells were further decreased compared to the sh‐NC‐R group, and the apoptosis rate was higher (*p* < 0.05, Figures 4f, 4g, and 4h). In other words, for both PLC/PRF/5 and PLC/PRF/5‐R, silencing *CLEC4G* expression can suppress cellular activity and promote apoptosis. Furthermore, when examining the status of the Wnt/*β*‐catenin pathway, it was observed that the protein expression levels of *β*‐catenin, *c-myc*, and *Cyclin-D1* in the sh‐CLEC4G group were lower compared with the sh‐RNA group (*p* < 0.05). Additionally, in the sh‐CLEC4G‐R group, the Wnt/*β*‐catenin pathway expression was also reduced compared to the sh‐RNA‐R group (*p* < 0.05, Figure 4i). These results suggest that silencing the expression of *CLEC4G* also exerts an inhibitory effect on the Wnt/*β*‐catenin pathway.

Figure 4Effect of *CLEC4G* on HCC. (a) Comparison of *CLEC4G* expression in cancerous and paracancerous tissues of HCC patients (*n* = 3). (b) Comparison of *CLEC4G* mRNA expression in peripheral blood of HCC patients before and after Lenva chemotherapy (*n* = 3). (c) Detection of cellular activity of PLC/PRF/5 and PLC/PRF/5‐R to determine drug resistance (*n* = 3). (d) Detection of *CLEC4G* mRNA expression to verify transfection success (*n* = 3). (e) IC_50_ of sh‐CLEC4G‐R group and sh‐RNA‐R group on Lenva (*n* = 3). (f) Cell cloning assay to detect the effect of *CLEC4G* on HCC cell activity (*n* = 3). (g) Transwell assay to detect the effect of *CLEC4G* on the invasive ability of HCC cells (*n* = 3). (h) Flow cytometry assay to detect the effect of *CLEC4G* on HCC cell apoptosis (*n* = 3). (i) Western blot to detect the effect of *CLEC4G* on Wnt/*β*‐catenin pathway (*n* = 3). *n* = 3 represents biological replicates (three independent experiments).  ^∗∗^ *p* < 0.01.

**Figure:**
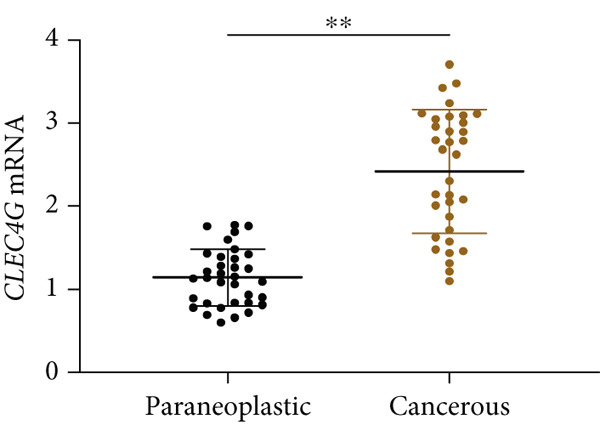
(a)

**Figure:**
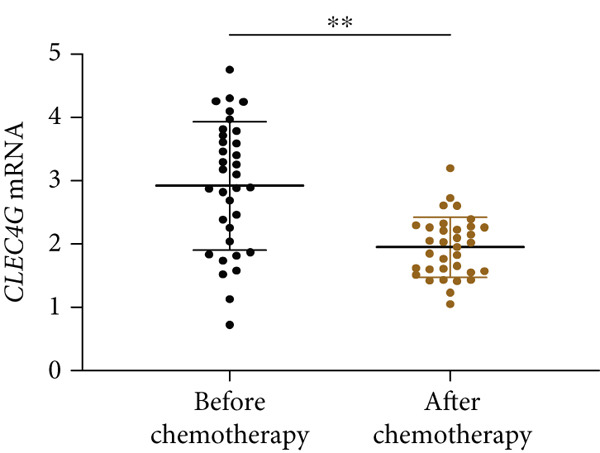
(b)

**Figure:**
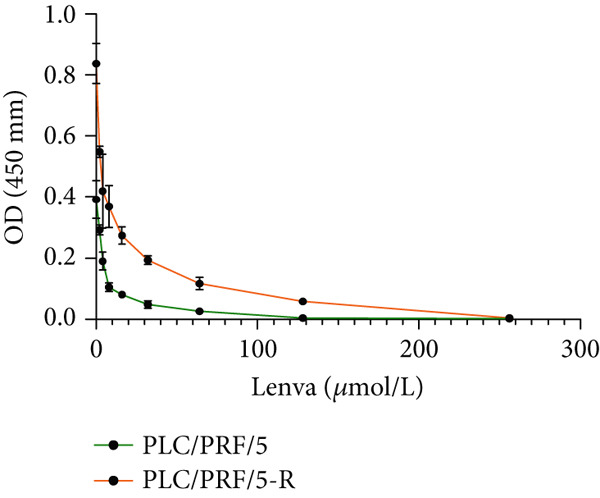
(c)

**Figure:**
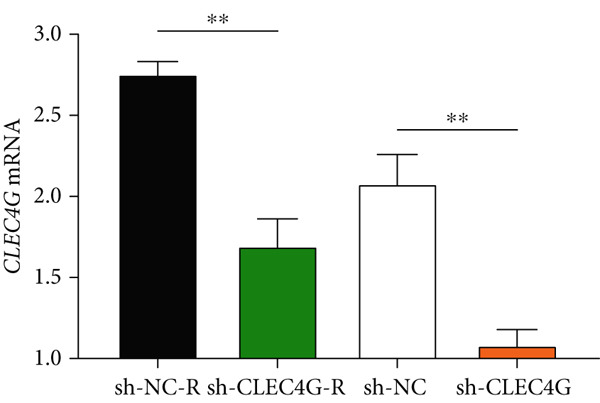
(d)

**Figure:**
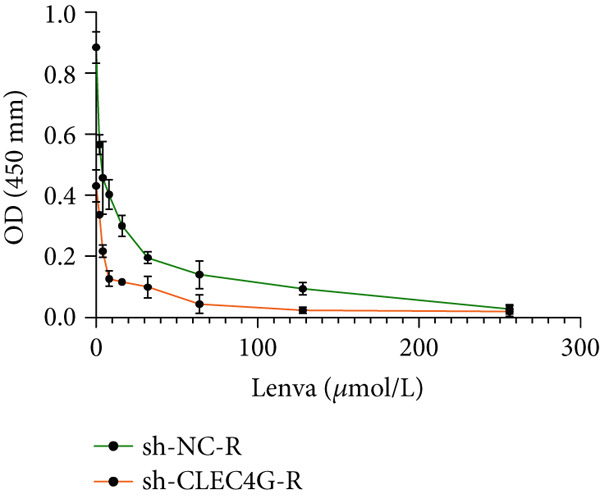
(e)

**Figure:**
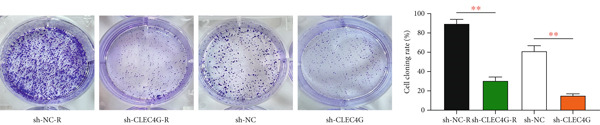
(f)

**Figure:**
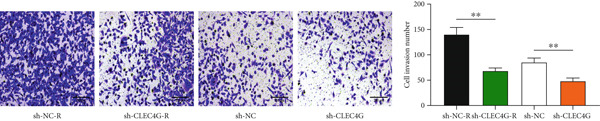
(g)

**Figure:**
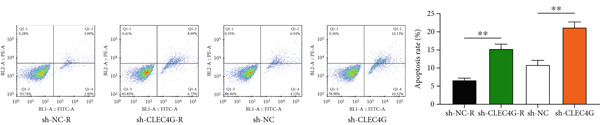
(h)

**Figure:**
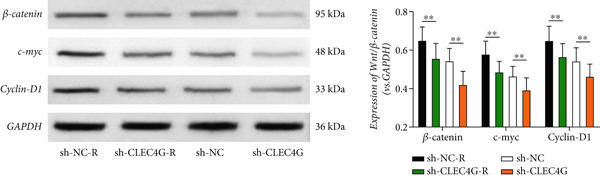
(i)

### 3.4. LiCl Activation of Wnt/*β*‐Catenin Reverses *CLEC4G*‐Mediated Effects

Based on the above, we have preliminarily identified that the impact of *CLEC4G* on HCC cells is associated with the Wnt/*β*‐catenin pathway. Consequently, we conducted further verification through a rescue experiment. First, through the intervention of LiCl, we found that *β*‐catenin, *c-myc*, and *Cyclin-D1* protein levels in the LiCl group exhibited a significant elevation in comparison to the control group (*p* < 0.05, Figure 5a), validating the success of the intervention. In terms of biological behavior, the proliferation rate and the invasive cell number of the LiCl group were markedly enhanced relative to the control group, whereas the apoptosis rate was decreased (*p* < 0.05, Figures 5b, 5c, and 5d), suggesting that the activation of the Wnt/*β*‐catenin pathway augments the activity of PLC/PRF/5‐R. In contrast, no significant differences were noted in the cell proliferation rate, invasive cell number, and apoptosis rate in the sh‐CLEC4G‐R + LiCl group when compared with the control group (*p* > 0.05, Figures 5b, 5c, and 5d), indicating that silencing *CLEC4G* expression can reverse the influence of LiCl. In the fluorescence staining test, it was noticed that, in comparison with the control group, the fluorescence intensity of *PD-1* in the sh‐CLEC4G‐R group was significantly diminished, while that in the LiCl group was elevated (*p* < 0.05, Figure 5e). This suggests that silencing *CLEC4G* inhibits the *PD-1* expression of cells, while activating the Wnt/*β*‐catenin pathway promotes this phenomenon. However, the fluorescence intensity of *PD-1* in the sh‐CLEC4G‐R + LiCl group showed no difference from that of the control group (*p* > 0.05, Figure 5e), reaffirming that *CLEC4G* can reverse the effect of the Wnt/*β*‐catenin pathway on cellular immune escape.

Figure 5
*CLEC4G* reverses the resistance of HCC cells to Lenva via the Wnt/*β*‐catenin pathway. (a) Detection of Wnt/*β*‐catenin pathway expression to determine the intervention effect of LiCl (*n* = 3). (b) *CLEC4G* affected the activity of HCC cells through the Wnt/*β*‐catenin pathway (*n* = 3). (c) *CLEC4G* affects the invasive ability of HCC cells through the Wnt/*β*‐catenin pathway (*n* = 3). (d) *CLEC4G* affects apoptosis of HCC cells through the Wnt/*β*‐catenin pathway (*n* = 3). (e) *CLEC4G* affects immune escape of HCC cells through the Wnt/*β*‐catenin pathway (*n* = 3). *n* = 3 represents biological replicates (three independent experiments).  ^∗∗^ *p* < 0.01.

**Figure:**
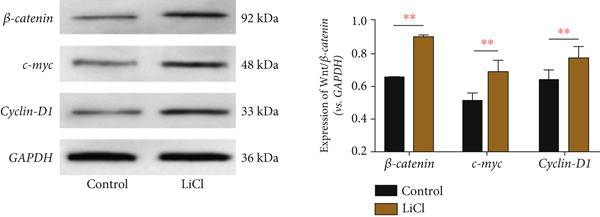
(a)

**Figure:**
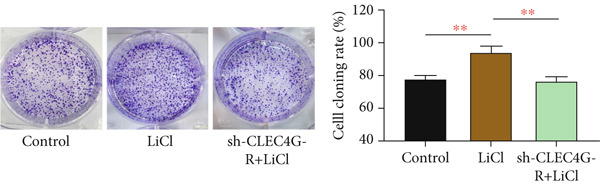
(b)

**Figure:**
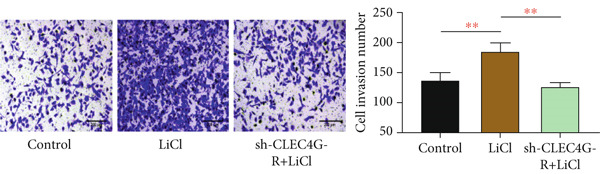
(c)

**Figure:**
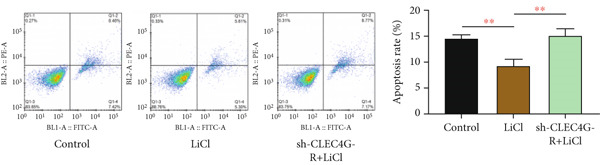
(d)

**Figure:**
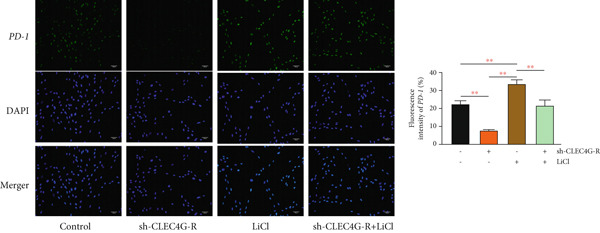
(e)

## 4. Discussion

Optimizing the diagnosis and treatment of HCC, as one of the major malignancies that endanger human life and safety, holds significant clinical value. In this research, we screened *CLEC4G*, a gene associated with autophagy in HCC, through an online database and discovered that it can effectively reverse the resistance of HCC cells to Lenva. These findings undoubtedly provide a more reliable therapeutic guarantee for the molecular‐targeted therapy of HCC in the future.

It has been well‐established in research that cellular immune escape is closely correlated with the occurrence and development of drug resistance [12, 13]. Hence, how to artificially modulate this process has emerged as the key to reversing cellular drug resistance. In this regard, researchers have proposed that intervening with DEGs in tumors might be a promising approach to achieve this goal [14]. Consequently, we conducted bioinformatics analysis to search for and select *CLEC4G* related to immune function in HCC as the primary research objective. Wang et al. also identified the key hub genes in HCC through bioinformatics analysis, including *CLEC4G* [15], which coincides with our findings. As a member of the C‐type lectin family, *CLEC4G* has been verified by previous studies to play a crucial role in the immune response. For instance, *CLEC4G* participates in atopic dermatitis progression by activating functional proteins related to the immune system [16]. In a single‐cell RNA‐sequencing analysis by Wang et al., *CLEC4G* has also been identified to be associated with alterations in the immune microenvironment of chronic sinusitis [17]. For HCC, although there is currently no research evidence supporting the role of *CLEC4G*, the study by Zhang et al. has mentioned that CLEC4S, a member of the *CLEC4G* family, is a therapeutic target in the immune microenvironment of HCC [18]. The results of biological information analysis in the current study showed that *CLEC4G* was significantly aberrantly expressed in HCC and was closely associated with the pathological staging of HCC. Compared with other cancers, CLEC4G expression in liver cancer shows a unique expression pattern: it is positively correlated with pathological stage in breast cancer and gastric cancer, but significantly affects the prognosis in lung cancer. This tissue specificity may result from the unique immunosuppressive network (such as Kupffer cell–dominated immune tolerance) in the HCC microenvironment, resulting in CLEC4G preferentially promoting immune escape through the Wnt/*β*‐catenin‐PD‐1 axis rather than directly driving proliferation. However, no obvious effect of *CLEC4G* on the prognosis of HCC was seen, which may be related to the small amount of data included in the current database. In contrast, the results of the clinical samples tested in this paper showed that the expression of *CLEC4G* was upregulated in cancer tissues, validating the results of the biological analysis described above. In immunoassays, we found that *CLEC4G* was associated with several immune checkpoints and regulatory genes in HCC and had a role in regulating CD4^+^ T cells and macrophages in HCC, and these results provide a reliable foundation for the idea that *CLEC4G* regulates immune escape in HCC.

In the above study, we obtained a preliminary understanding of the expression of CLEC4G in HCC. Nevertheless, hitherto, no further research has been carried out to verify precisely the effect of *CLEC4G* on HCC. Our study found that the expression of *CLEC4G* was decreased after Lenva chemotherapy, which suggested a potential association between *CLEC4G* and Lenva. Therefore, to examine the impact of *CLEC4G* on the drug resistance of HCC, we intervened in the expression of *CLEC4G* in cells by transfecting abnormal expression vectors. It was found that silencing *CLEC4G* expression effectively inhibited PLC/PRF/5 and PLC/PRF/5‐R activity and promoted apoptosis. These results initially validate the regulatory effect of *CLEC4G* on the biological behavior of HCC cells. Furthermore, for Lenva‐resistant cells, the activity‐inhibitory effect demonstrated by *CLEC4G* offers novel prospects for reversing the drug resistance of molecular‐targeted drugs in the future. Previous studies regarding *CLEC4G* have commonly focused on the relationship between *CLEC4G* and inflammatory responses. For example, *CLEC4G* has been identified as a novel inflammatory biomarker related to the severity of stroke [19]. Besides, it functions as an inflammatory checkpoint in psoriatic skin tissue [20]. We hold the view that the regulatory effect of *CLEC4G* on inflammatory responses, which has been frequently alluded to in previous studies, is also attributable to its substantial impact on the immune microenvironment. Research has indicated that *CLEC4G* can activate M2‐type macrophages, thus facilitating the immune escape of lung cancer cells [21]. Similarly, the research by Zhang Y et al. suggests that *CLEC4G* and Rab1A, two transmembrane proteins highly expressed in macrophages, are the main binding targets of AELP‐B6. They are co‐located with the cell membrane and have a direct impact on the activation of immune cells and apoptosis [22]. These results fully demonstrate the important role of *CLEC4G* in the cellular immune microenvironment, which can produce a cascade reaction to promote a series of biological behavior changes such as inflammation and fibrosis. That is to say, in the future, targeted silencing of *CLEC4G* expression can not only reverse the drug resistance of HCC cells to Lenva but also further promote the death of tumor cells, thus providing a more reliable guarantee for the treatment and prognosis of HCC patients.

To further clarify the mechanism of the action of *CLEC4G* on HCC, we carried out further research to find the action pathway. Referring to previous studies, it was found that the signal transduction pathway involved in *CLEC4G* is mainly related to Wnt/*β*‐catenin [23], one of the most classic signal pathways in clinical research. The association between Wnt/*β*‐catenin and HCC has been verified multiple times [24, 25]. However, currently, there is no research to confirm whether the impact of *CLEC4G* on HCC is related to the Wnt/*β*‐catenin pathway. C‐Myc and Cyclin‐D1 are key downstream effectors of the Wnt/*β*‐catenin pathway, and their expression changes can directly reflect the activity of the Wnt/*β*‐catenin pathway [26]. In this study, we found that silencing *CLEC4G* expression could suppress the state of the Wnt/*β*‐catenin pathway in PLC/PRF/5 and PLC/PRF/5‐R. Additionally, upon using LiCl to activate the Wnt/*β*‐catenin pathway expression in PLC/PRF/5‐R, we observed an enhancement in cell activity and a reduction in apoptosis. This is in line with the results of previous studies [27], reaffirming that the activated Wnt/*β*‐catenin pathway promotes the progression of HCC. Research shows that the Wnt/*β*‐catenin pathway is a highly conserved and strictly controlled molecular mechanism that can regulate embryonic development, cell proliferation, and differentiation [28]. In many tumor diseases, including HCC, the activation of the Wnt/*β*‐catenin pathway can augment various biological behaviors such as tumor cell differentiation and glycolysis, thereby facilitating tumor growth [29, 30]. Similarly, in investigations concerning the immune microenvironment of HCC cells, mutations in the Wnt/*β*‐catenin pathway are also regarded as key to reversing immune escape [31]. These studies have established a solid foundation for the potential connection between *CLEC4G* and the Wnt/*β*‐catenin pathway. Through the rescue experiment, we found that silencing *CLEC4G* expression could completely reverse the influence of LiCl on PLC/PRF/5‐R, confirming that the effect of *CLEC4G* on HCC is associated with the Wnt/*β*‐catenin pathway. Furthermore, in fluorescence staining, we observed the effects of *CLEC4G* and Wnt/*β*‐catenin on the expression of *PD-1* in PLC/PRF/5‐R, which once again proves that *CLEC4G* modulates the immune escape behavior of HCC through the Wnt/*β*‐catenin pathway. The study of Azzahra et al. mentioned that *CLEC4G*, as a C‐type lectin receptor, may directly bind Wnt ligands (e.g., Wnt3a) and promote *β*‐catenin nuclear translocation [32]. Meanwhile, *CLEC4*G may enhance signaling by modulating the glycosylation status of Wnt receptors (e.g., frizzled) [33]. These findings suggest a regulatory mechanism of *CLEC4G* for the Wnt/*β*‐catenin pathway, which will be further verified in subsequent studies using Co‐IP and glycosylation inhibitors. At the same time, CLEC4G may be used as a dynamic marker to predict the efficacy of Lenva: patients with high expression before treatment may need to combine with Wnt inhibitors (such as ICG‐001), and those with sustained high expression after chemotherapy may indicate the risk of drug resistance.

Nevertheless, considering the limitations of the research conditions, more in‐depth research is required to ascertain the influence of *CLEC4G* on HCC and its relation to the Wnt/*β*‐catenin pathway. We plan to resolve the dynamics of immune cell subsets and cytokines in the *CLEC4G*‐deficient microenvironment by single‐cell sequencing. Meanwhile, it is essential to conduct in vivo animal experiments to observe the effect of *CLEC4G* on the actual tumor growth of HCC. Although the effect of CLEC4G on the invasive ability of HCC cells was evaluated by Transwell assay in this study, the direct validation of the migration phenotype needs to be further explored. In the future, we plan to clarify the role of CLEC4G in regulating cell migration by scratch assay. At the same time, the effect of CLEC4G on cell cycle distribution (such as G1/S phase arrest) needs to be detected by PI staining flow cytometry. Finally, PD‐1 down‐regulation may enhance the synergistic effect of PD‐1 inhibitors (such as pembrolizumab) with Lenva, and phase II clinical trials are recommended to verify the OS benefit. Although bioinformatics suggested that CLEC4G was associated with CD4+ T cell and macrophage infiltration, the experimental data of this study were not sufficient to support a strong conclusion of “synergy.” Current evidence can be summarized into three levels: Direct evidence: CLEC4G silencing reduces PD‐1 expression, which serves as a marker of T‐cell exhaustion and its downregulation may restore antitumor immune responses. Indirect evidence: LiCl increased the expression of PD‐1 after activating the Wnt pathway, and this effect could be reversed by CLEC4G silencing, confirming the existence of the CLEC4G‐Wnt‐PD‐1 axis. Predictive evidence: The TIMER database showed that CLEC4G copy number variation was positively associated with M2 polarization of macrophages, but SCNA analysis did not show a significant effect on immune infiltration. Therefore, it is necessary to quantify the changes in the proportion of CD8+ T cells (such as CXCR3 + CD8+ T cells) after CLEC4G silencing by flow cytometry in future studies. At the same time, a humanized HCC mouse model was established to verify whether CLEC4G deletion enhanced the efficacy of the PD‐1 inhibitor.

## 5. Conclusion

*CLEC4G* modulates the *PD-1* expression of HCC cells via the Wnt/*β*‐catenin pathway, thus reversing the resistance to Lenva. The downregulation of *PD-1*, an important immune checkpoint molecule, may be associated with the regulation of the immune microenvironment. This offers a novel intervention approach for the targeted therapy of HCC in the future, enabling the enhancement of the treatment efficacy of Lenva and the improvement of the outcomes of HCC patients. However, more experiments are needed to realize its clinical application.

## Disclosure

All authors read and approved the final submitted manuscript.

## Conflicts of Interest

The authors declare no conflicts of interest.

## Author Contributions

G.H. conceived and designed the study. K.X. and J.Y. wrote and revised the manuscript. B.H. collected and analyzed data. K.X. and J.Y. made equal contributions in this work as co‐first authors. K.X. and J.Y.′s contributions in this work are equal.

## Funding

No funding was received for this manuscript.

## Data Availability

The data that support the findings of this study are available from the corresponding author upon reasonable request.
